# Examining the impact of sex differences and the COVID-19 pandemic on health and health care: findings from a national cross-sectional study

**DOI:** 10.1093/jamiaopen/ooac076

**Published:** 2022-09-28

**Authors:** Jiancheng Ye, Zhimei Ren

**Affiliations:** Feinberg School of Medicine, Northwestern University, Chicago, Illinois, USA; Department of Statistics, University of Chicago, Chicago, Illinois, USA

**Keywords:** multivariate model, multiple hypothesis testing, sex differences, patient-generated health data, health disparity, health impact, COVID-19 pandemic

## Abstract

**Objective:**

To examine the association of the coronavirus disease 2019 (COVID-19) pandemic, the association of sex, and the joint association of sex and the COVID-19 pandemic with health communication, physical activity, mental health, and behavioral health.

**Materials and Methods:**

We drew data from the National Cancer Institute's 2020 Health Information National Trends Survey. We described and compared the characteristics of social determinants of health, physical activity, mental health, alcohol use, patterns of social networking service use, and health information data sharing. Analyses were weighted to provide nationally representative estimates. Multivariate models (multiple linear regression, multiple logistic regression, and multinomial logistic model) were used to assess the sole and joint association with sex and pandemic. In addition, we applied the Bonferroni correction to adjust *P* values to decrease the risks of type I errors when making multiple statistical tests.

**Results:**

Females were more likely to use mobile health and health communication technologies than males, and the difference increased after the pandemic. The association between sex and mental health was significant after the COVID-19 pandemic. Females were more likely to experience depression or anxiety disorders. Both males and females had a slight decrease in terms of the quantity and intensity of physical activity and females were less likely to perform moderate exercise and strength training regularly. Males were likely to drink more alcohol than females.

**Conclusion:**

The COVID-19 pandemic amplifies the differences between males and females in health communication, physical activity, mental health, and behavioral health. Intersectional analyses of sex are integral to addressing issues that arise and mitigating the exacerbation of inequities. Responses to the pandemic should consider diverse perspectives, including sex and gender.

## INTRODUCTION

The coronavirus disease 2019 (COVID-19) pandemic has uprooted conventional health care delivery for various care, such as primary care and mental health care, requiring health systems to rapidly adopt new capabilities.^1^^,^^2^ With mandated social distancing policies, health care providers have gotten closely acquainted with virtual health.^3^ Virtual and remote care offers benefits such as accessibility. The additional benefits for both patients and providers can come from effectively leveraging patient-generated health data during these visits.^4^ However, evidence shows mixed results regarding whether the widespread transition to telehealth during the pandemic is creating additional fractures in society by disproportionately harming health equity.^5^ The COVID-19 pandemic has further exacerbated inequities in health care, including patients’ access to necessary care and relevant health information.^6^ There are also sex differences in health information technology acceptability, physical activity, and mental health.^7^ The digital divide has shown lower rates of technology and broadband adoption among lower socioeconomic statuses.^8^^,^^9^

Gender is defined as a social construction that assigns characteristics, norms, and roles of women and men according to social norms; gender represents the different roles that society expects of people.^10^ The sex assigned to a child at birth is most often based on the child's external anatomy; sex is also referred to as birth sex, natal sex, or biological sex.^10^ While sex is described as female, male, and intersex, gender can be described as feminine, masculine, androgynous, and much more.^10^ Regardless of the pandemic, females on average have reported more challenging physical and mental unhealthy status than males.^11^ The pandemic has differential impacts on females and males from the risks of exposure and biological susceptibility to infection, social and economic implications, and individuals’ experiences. These impacts are likely to vary according to biological and sex characteristics and their interaction with other social determinants.^12^ It is warranted to understand the impact so that targeted health care delivery strategies can be implemented.^13^ Failing to consider sex differences in COVID-19 research may negatively impact the effectiveness of the responses to the pandemic and possibly increase health inequities.^14^ Recognizing the extent to which the pandemic affects females and males differently is a fundamental step to understanding the effects of the global public health emergency on different individuals and communities, and creating effective interventions and equitable policies.^15^

The COVID-19 pandemic has had unprecedented and potentially irreversible impacts on health and health care globally with ongoing and adverse impacts. There are therefore many complex issues and factors that need to be accounted for as we look at the long-term impact of COVID-19 on humanity and society and how this ongoing crisis continues to affect health and health care for different groups. Sex-responsive interventions are imperative to address widening sex gaps resulting from the pandemic. Although many researchers have studied the impact of COVID-19 on health disparities,^16^ there is a lack of research on the sole and joint association of COVID-19 or sex differences and their specific impact on health and health care during the pandemic. The aim of this study is to examine the association of the COVID-19 pandemic, the association of sex, and the joint association of sex and the COVID-19 pandemic with health communication, physical activity, mental health, and behavioral health.

## METHODS

### Study design

Data for this study were drawn from the National Cancer Institute’s 2020 Health Information National Trends Survey (HINTS). HINTS is a nationally representative survey administered every year by the National Cancer Institute, which provides a comprehensive assessment of the American public’s current access to and use of health information.^17^ The target population of HINTS is civilians, noninstitutionalized adults aged 18 or older living in the United States. In this study, we aim to examine the association of the COVID-19 pandemic, the association of sex, and the joint association of sex and the COVID-19 pandemic with health communication, physical activity, mental health, and behavioral health. We utilized the birth sex in the HINTS to indicate the sex of the participants. We transformed the continuous variable of age into a categorical variable by classifying age into 4 groups: (1) 18–34 years, (2) 35–49 years, (3) 50–64 years, and (4) ≥65 years. Education level was recategorized as a high school diploma or less, some college, and college graduate or higher. Annual income level was categorized as ≤$20 000, $20 000–$35 000, $35 000–$50 000, $50 000–$75 000, and >$75 000. The variables related to health communication, physical activity, mental health, and behavioral health were based on the multiple-choice questions in each of the corresponding sections of HINTS. The hot-deck imputation was used to handle missing data.

### Study participants

Data used in this study were from the third round of data collection for HINTS 5 (Cycle 4), which began in February 2020 and concluded in June 2020. All data referred to in the manuscript are available at: https://www.cancer.gov/ and the Dryad Digital Repository: doi:10.5061/dryad.h70rxwdn5. A binary variable indicating whether the participants returned their survey before or after the COVID-19 pandemic was provided, determined by the World Health Organization’s announcement on March 11 of the COVID-19 pandemic. This variable facilitates the examination of responses before and after COVID-19 in the United States. The final HINTS 5, Cycle 4 (2020) sample consists of 3865 respondents.

### Model

To understand the association of the pandemic, the association of sex, and the joint association of sex and pandemic with the health care domains (health communication, physical activity, mental health, and behavioral health), we characterize the response of interest via a multivariate model.^18^ Specifically, If *Y* is continuous, we assume it to be generated from the following linear model:

(Model 1) $$Y=\beta_{0}+\beta_{1}\cdot \mathrm{Pandemic}\;+\beta_{2}\;\cdot \mathrm{Sex}\;+\gamma \cdot \mathrm{Pandemic}\cdot \mathrm{Sex}\;+\alpha_{1}\;\cdot \mathrm{Age}\;+\alpha_{2}^{T}\cdot \mathrm{Ethnicity}+\alpha_{3}^{T}\cdot \mathrm{Ethnicity}+\alpha_{4}^{T}\cdot \mathrm{Marital}\;\mathrm{status}\;+\alpha_{5}\cdot \mathrm{Edcuation}+\alpha_{6}\cdot \mathrm{Income}+\varepsilon \;.$$

The *Pandemic* is a dichotomous variable indicating whether the response was obtained before or after the declaration of the pandemic; *Sex* is a dichotomous variable with 0 referring to the male and 1 to the female; ε is a zero-mean normal random variable. Sociodemographic variables are incorporated in the model to account for possible confounding factors, where *Age* and *Income* are treated as continuous variables; *BMI, Ethnicity*, *Education Marital Status* are categorical variables. In model 1, $\beta_{1}\;$is the difference between the mean of the response after and before the pandemic for the male populations (adjusting the demographic covariates); $\beta_{1}+\gamma$ is the same difference but in the female population. $\beta_{2}$ represents the difference in the mean of the responses between females and males (adjusting the demographic covariates) before the pandemic, and $\beta_{2}+\gamma$ is the difference after the pandemic. The coefficient γ stands for the change of the sex difference before and after the pandemic, that is,$\;\left(\beta_{2}+\gamma \right)-\beta_{2}$.

When the response *Y* is dichotomous (*Y* = 0 is the reference), we build model 2, where we assume *Y* to be generated from a logistic regression model:

(Model 2) $$\log(\frac{P\left(Y=1\;\right|\;\mathrm{Covariates})}{P\left(Y=0\;\right|\;\mathrm{Covariates})})\;=\beta_{0}+\beta_{1}\cdot \mathrm{Pandemic}+\beta_{2}\cdot \mathrm{Sex}+\gamma \;\cdot \mathrm{Pandemic}\cdot \mathrm{Sex}\;+\alpha_{1}\cdot \mathrm{Age}+\alpha_{2}^{T}\;\cdot \mathrm{Ethnicity}+\alpha_{3}^{T}\cdot \mathrm{Ethnicity}+\alpha_{4}^{T}\cdot \mathrm{Marital}\;\mathrm{status}+\alpha_{5}\cdot \mathrm{Edcuation}+\alpha_{6}\cdot \mathrm{Income}.$$

In model 2, the exp⁡(β_1_) is the odds ratio (OR) of the male population after and before the pandemic adjusting the demographic covariates, and the $\exp(\beta_{1}+\;\gamma )$ is for the female population. The exp (β_2_) stands for the OR between females and males before the pandemic, and $\exp(\beta_{2}+\;\gamma )$ is the OR between females and males after the pandemic. The exp (γ) is the ratio of the sex odds ratio after and before the pandemic, that is,$\;\exp(\beta_{1}+\;\gamma )/\exp(\beta_{2})$.

When the response *Y* is a categorical variable with more than 2 classes, we consider a multinomial logistic regression model.^19^ Supposing the categories of the *Y* are denoted by the set= {1, 2, …, *K*}, we set the first category as the baseline, for each k=2, …, K and build model 3:

(Model 3) $$\log(\frac{P\;\left(Y=k\;\right|\;\mathrm{Covariates})}{P\;\left(Y=0\;\right|\;\mathrm{Covariates})})\;=\beta_{k,0}+\beta_{k,1}\cdot \mathrm{Pandemic}+\beta_{k,2}\cdot \mathrm{Sex}\;+\gamma_{k}\cdot \mathrm{Pandemic}\cdot \mathrm{Sex}+\alpha_{k,1}\cdot \mathrm{Age}\;+\alpha_{k,2}^{T}\cdot \mathrm{Ethnicity}+\alpha_{k,3}^{T}\cdot \mathrm{Ethnicity}+\alpha_{k,4}^{T}\cdot \mathrm{Marital}\;\mathrm{status}+\alpha_{k,5}\cdot \mathrm{Edcuation}+\alpha_{k,6}\cdot \mathrm{Income}.$$

The interpretation of model 3 is the same as model 2 except that we are comparing category *k* to the reference category.

### Statistical analysis

Statistical analyses were performed using R version 4.0.5 (R Foundation, Vienna, Austria). For continuous responses, ordinary least squares estimators of the coefficients in the linear model are estimated with the lm function in R and reported with the *P* values; for categorical responses, adjusted odds ratios (aORs) are estimated with the glm package in R, and reported with the *P* values. The *P* values for the sum of 2 coefficient estimators are obtained using the multcomp package in R, and all statistical testing was 2-tailed. In this study, 50 characteristics were categorized into 4 health care domains (health communication, physical activity, mental health, and behavioral health). Within each domain, there were 5 hypotheses to be tested: the association of sex before the pandemic, the association of sex after the pandemic, the association of the pandemic in the male population, the association of the pandemic in the female population, and the interacting association of sex and pandemic with the 4 domains.

Commonly, *P* = .05 is used as the significance threshold for a single hypothesis testing. In this study, 50*5 = 250 hypotheses are tested simultaneously. Therefore, it is necessary to adjust for the multiplicity of the hypotheses, to avoid type I errors.^20^ Assuming that all the 250 hypotheses are null, if we are still using the 0.05 threshold, then the probability of making at least a false discovery is the probability of at least 1 *P* value exceeding .05. When the *P* values are mutually independent, the value is 1−(0.95) ^ 250 ≈ 1.00, which means we almost surely make at least 1 false discovery. Meanwhile, the expected number of discoveries is 250*0.05 = 12.5, and these discoveries are all, by definition, false discoveries. To address this issue, we need to adjust the *P*-value threshold based on the number of hypotheses being tested. In particular, we applied the Bonferroni correction^21^ to adjust *P* values to decrease the risks of type I errors, which proceeded as the following steps: when testing m hypotheses simultaneously, a *P*-value threshold of .05/m was utilized. With the adjusted threshold, the $m_{0}$ denotes the number of null hypotheses, and the probability of making at least 1 false discovery can be bounded as:

$$p\;\left(\;\underset{i:\mathrm{hypothesis}\;i\;\mathrm{is}\;\mathrm{null}}{⋃}p_{i}\;\leq \frac{0.05}{m}\right)\;\leq \;\sum_{i=1}^{m_{0}}p\;\left(\;p_{i}\;\leq \frac{0.05}{m}\right)\;\leq \;\frac{m_{0}}{m}\;0.05\;\leq \;0.05.$$

The above derivation illustrates that the probability of making at least 1 false discovery is no more than 0.05, which is desirable. As a result, a *P* value < .05/250 = .0002 is designated as statistically significant in our statistical analyses. We indicated the significant variables in the results section and all the *P* values for Supplementary Tables S2–S4 and Figure S1 are shown in the supplemental tables.

## RESULTS

Table 1 demonstrates the sociodemographic characteristics of the participants in this study. We present the weighted and unweighted prevalence of subcategories for each variable, and the weighted prevalence of the subcategories before and after the COVID-19 pandemic. There were more younger participants and white participants after the pandemic, but the other sociodemographic covariates did not show significant differences.

**Table 1.** Unweighted and weighted prevalence estimates for sample sociodemographic, Health Information National Trends Survey (HINTS) 5 Cycle 4 (2020)

Characteristics	Overall unweighted, %	Overall weighted, %	Before pandemic, weighted, %	After pandemic, weighted, %	*P* value
Age, %					<0.001
18–34	12.9	26.2	19.9	29.6	
35–49	18.8	25.6	22.7	27.1	
50–64	30.6	27.7	33.0	24.8	
65+	37.6	20.5	24.4	18.4	
Male, %	41.5	48.6	51.0	47.4	0.27
BMI, %					0.36
Underweight	1.7	1.3	1.4	1.2	
Normal weight	31.6	34.1	33.0	34.6	
Overweight	33.7	31.5	34.0	30.1	
Obesity	33.1	33.2	31.6	34.1	
Race/ethnicity, %					<0.001
Non-Hispanic White	61.3	63.4	70.1	59.8	
Non-Hispanic Black or African American	13.7	11.0	8.1	12.6	
Hispanic	16.9	16.9	13.0	19.1	
Non-Hispanic Asian	4.7	5.3	4.2	5.8	
Non-Hispanic Other	3.4	3.3	4.6	2.7	
Marital status, %					0.17
Married	48.7	50.6	54.5	48.6	
Divorced	16.1	8.3	8.2	8.4	
Widowed	11.0	4.6	5.2	4.3	
Other^a^	24.2	36.4	32.1	38.8	
Education, %					0.15
High school diploma or less	33.2	39.2	38.2	39.8	
Some college	21.9	30.3	28.4	31.3	
College graduate or higher	44.9	30.5	33.4	28.9	
Income, %					0.58
Less than $20 000	17.8	14.9	13.6	15.5	
$20 000–$34 999	13.0	11.4	10.5	11.8	
$35 000–$49 999	13.4	12.7	12.2	13.0	
$50 000–$74 999	17.3	18.4	17.8	18.8	
$75 000 or more	38.6	42.7	45.9	40.9	

a Other includes single (never been married), separated, living as married or living with a romantic partner.

Table 2 presents the estimates of the multivariate model in terms of the variables corresponding to health communication. To compare the association of sex and health communication variables before or after the pandemic, the female is the reference group; to compare the association of pandemic and health communication variables in the male or female group, “before pandemic” is the reference group. For the characteristic *made appointments with a health care provider online*, the association with sex before the pandemic is not significant but becomes significant after the pandemic, where the aOR between the female and male is e^−0.36^ = 0.70 (the other aORs in the tables can be interpreted in the same way), which means the odds of making appointments with a health care provider online for males is 0.70 times that of females; the association with the pandemic is not significant either before or after the pandemic, and the interacting association with the pandemic and sex is not significant either. As for the characteristic *looked for health or medical information for oneself*, the association with sex is significant before the pandemic, with an aOR = e^−0.65^ = 0.52, which means the odds of looking for health or medical information for males is 0.52 times of the female; the association with sex is also significant after the pandemic, with an aOR between the female and male groups is e^−0.55^ = 0.58.

**Table 2.** Multivariate modeling of the association of COVID-19 pandemic, the association of sex, and the joint association of sex and COVID-19 pandemic in relation to health communication

Characteristics	Sex (before pandemic), aOR or coefficient	Sex (after pandemic), aOR or coefficient	Pandemic (male), aOR or coefficient	Pandemic (female), aOR or coefficient	Sex*Pandemic, aOR or coefficient
Made appointments with a health care provider online	−0.21	−0.36*	−0.06	−0.20	−0.14
Looked for health or medical information	−0.65*	−0.55*	−0.01	0.09	0.10
Used e-mail or the Internet to communicate with doctors	−0.30	−0.12	−0.27	−0.09	0.17
Used electronic means to look up medical test results	−0.47*	−0.38*	−0.01	0.09	0.10
Frequency of using a wearable device to track health					
Every day	Ref	Ref	Ref	Ref	Ref
Almost every day	0.10	0.02	−0.17	−0.26	−0.09
1– times per week	−0.53	0.34	−0.91	−0.03	0.88
Less than once per week	−0.29	0.11	0.28	0.68	0.40
Not use a wearable device	−0.04	0.50	−0.54	0.002	0.54
Shared health information on social networking sites	−1.14*	−0.70*	−0.47	−0.03	0.44
Participated in an online forum or support group	−1.53*	−0.77*	−0.72	0.03	0.75
Visited a social networking site	−0.81*	−0.50*	−0.41	−0.10	0.31
Watched a health-related video	−0.34	−0.06	−0.32	−0.04	0.28
Shared health information from either an electronic monitoring device or smartphone	0.001	−0.04	−0.09	−0.13	−0.04
Used smartphone to track progress on a health-related goal	−0.49*	−0.46*	0.03	0.06	0.03
Used smartphone to discuss with your health care provider	−0.19	−0.30*	−0.01	−0.11	−0.10
Used smartphone to make a decision about how to treat an illness or condition	−0.32	−0.16	−0.04	0.13	0.16
Had “apps” related to health and wellness	−0.42*	−0.39*	−0.10	−0.07	0.03
Used health or wellness apps	−0.44	−0.41	0.05	0.09	0.03
Used wearable devices to monitor or track health or activity	−0.54*	−0.56*	0.003	−0.02	−0.02
Willing to share health data from wearable device with health care provider	0.20	−0.004	0.22	0.02	−0.20
Willing to share health data from wearable device with family	0.30	−0.06	0.35	−0.01	−0.36
Willing to share health data from wearable device with friends	−0.22	−0.38	0.002	−0.16	−0.16

* *P* < .0002.

aOR: adjusted odds ratio; COVID-19: coronavirus disease 2019.

To interpret the results comprehensively, we provide an example of the characteristics *used electronic means to look up medical test results,* the association with sex is significant before the pandemic, with an aOR = e^−0.47^ = 0.63, meaning that the odds of looking up medical results in males is 0.63 times of females; the association with sex is also significant after the pandemic, with an aOR between the female and male groups is e^−0.38^ = 0.68. The aOR between before and after the pandemic in the male group is e^−0.01^ = 0.99, meaning the odds of using electronic means to look up medical results before the pandemic is 0.99 times of those after the pandemic in the male group while the association is not significant; the corresponding aOR in the female group is e^0.09^ = 1.09, meaning the odds of using electronic means to look up medical results before the pandemic is 0.99 times those after the pandemic in the female population, the association is not significant either. The last column represents the change in the odds ratio between sexes before and after the pandemic. Specifically, the aOR between males and females after the pandemic is e^0.10^ = 1.11 times that before the pandemic, while this association is not significant.

Similarly, we see a significant association with sex both before and after the pandemic in characteristics including *shared health information on social networking sites*, *participated in an online forum or support group*, *visited social networking sites*, *used smartphone to track progress on a health-related goal* and *used an electronic wearable device to monitor or track health or activity.* We also see a significant association with sex after the pandemic with regard to the characteristic *have “apps” related to health and wellness.* We see sex differences in using mobile health and social networking services and females are more likely to use these tools while the differences are not statistically amplified by the pandemic.

Table 3 presents the model estimates for the characteristics corresponding to mental health. For *Depression or Anxiety*, the association with sex is significant before and after the pandemic, with estimated associations being −0.09 and −0.13, respectively. This means the difference in the levels of having depression or anxiety disorder in females and males are −0.09 and −0.13 before and after the pandemic, respectively. For *Feeling nervous, anxious, or on edge*, the association with sex is significant before and after the pandemic, with an estimated coefficient of −0.15 and −0.19, respectively, suggesting that females are more likely to feel nervous or anxious. The association with sex demonstrates a significant association for *not being able to stop or control worrying* after the pandemic, with an expected difference of −0.19. Sex also shows a significant association with characteristics like *When I feel threatened or anxious, I find myself thinking about my strengths* before the pandemic, with an expected difference of −0.17. The characteristic *Most important value* is categorical with more than 2 classes. As introduced in the “Methods” section, we set *Making my own decisions* as the reference, and compare other values to it. The association with sex is significant after the pandemic for the characteristics *being happy, having a deep connection to my religion and assuring my family is safe and secure* (when compared with the reference), and the aORs are 3.53 and 1.93, respectively. Overall, females are more possible to have mental health issues.

**Table 3.** Multivariate modeling of the association of COVID-19 pandemic, the association of sex, and the joint association of sex and COVID-19 pandemic in relation to mental health

Characteristics	Sex (before pandemic), aOR or coefficient	Sex (after pandemic), aOR or coefficient	Pandemic (male), aOR or coefficient	Pandemic (female), aOR or coefficient	Sex*Pandemic, aOR or coefficient
General health	−0.003	0.02	0.02	0.04	0.02
Confident about ability to take good care of health	−0.04	−0.06	0.05	0.03	−0.02
Depression or anxiety disorder	−0.09*	−0.13*	0.01	−0.02	−0.03
Little interest or pleasure in doing things	−0.01	−0.01	0.003	−0.001	−0.004
Feeling down, depressed, or hopeless	−0.11	−0.06	−0.03	0.01	0.05
Feeling nervous, anxious, or on edge	−0.15*	−0.19*	−0.02	−0.06	−0.04
Not being able to stop or control worrying	−0.23	−0.19*	−0.02	0.01	0.03
When I feel threatened or anxious, I find myself thinking about my values	−0.14	−0.08	−0.10	−0.04	0.06
When I feel threatened or anxious, I find myself thinking about my strengths	−0.17*	−0.06	−0.14	−0.03	0.11
Most important value					
Making my own decisions	Ref	Ref	Ref	Ref	Ref
Being happy	0.01	0.54	−0.44	0.09	0.53
Helping people	0.40	0.59	−0.01	0.17	0.19
Being loyal to family and friends	0.23	−0.19	−0.13	−0.56	−0.43
Having a deep connection to my religion	0.46	1.26*	−0.45	0.35	0.80
Keeping myself in good health	0.08	0.40	−0.17	0.15	0.32
Assuring my family is safe and secure	0.03	0.66*	−0.21	0.42	0.63

* *P* < .0002.

aOR: adjusted odds ratio; COVID-19: coronavirus disease 2019.

Table 4 presents the model estimates for variables corresponding to behavioral health. In this table, the *Number of days drinking alcohol* and *Average drinks per day* are treated as continuous variables, and *Frequency of alcohol use per month* is treated as a categorical variable. For the number of days drinking alcohol, the association with sex is significant before and after the pandemic, with estimated coefficients of −0.59 and −0.39, respectively. Males were likely to drink more alcohol than females. Similarly, there is a significant association with sex for *average drinks per day*, before and after the pandemic. The characteristic *Frequency of alcohol use per month* is a categorical variable with 5 classes. We set *Never* as the reference and compare other classes to the reference, and there are no significant associations either with sex or the pandemic.

**Table 4.** Multivariate modeling of the association of COVID-19 pandemic, the association of sex, and the joint association of sex and COVID-19 pandemic in relation to behavioral health

Characteristics	Sex (before pandemic), aOR or coefficient	Sex (after pandemic), aOR or coefficient	Pandemic (male), aOR or coefficient	Pandemic (female), aOR or coefficient	Sex*Pandemic, aOR or coefficient
No. of days drinking alcohol	−0.59*	−0.39*	−0.14	0.06	0.20
Average drinks per day	−0.64*	−0.67*	−0.18	−0.20	−0.03
Frequency of alcohol use per month					
Never	Ref	Ref	Ref	Ref	Ref
1–2	−0.35	−0.24	0.03	0.14	0.11
3–5	−0.20	−0.62	0.37	−0.05	−0.42
6–10	−0.78	−0.39	−0.39	−0.01	0.39
11+	−0.83	−0.18	−0.21	0.44	0.65

* *P* < .0002.

aOR: adjusted odds ratio; COVID-19: coronavirus disease 2019.

**Figure 1.**
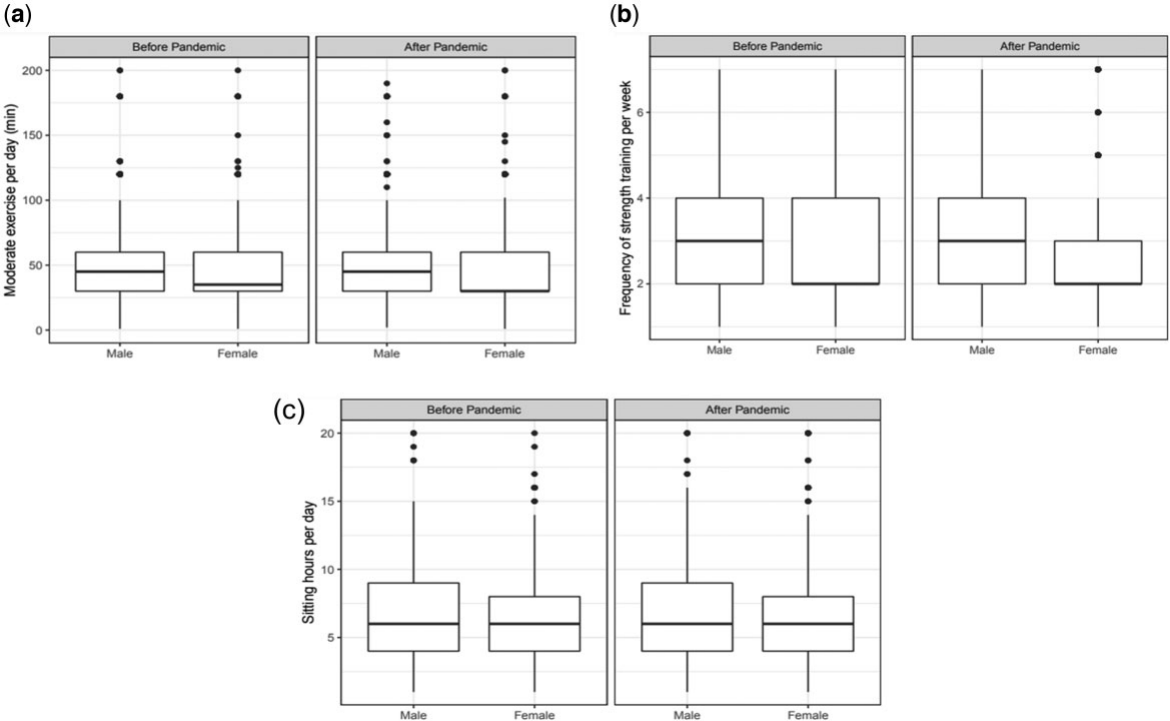
Multivariate modeling of the association of the COVID-19 pandemic, the association of sex, and the joint association of sex and COVID-19 pandemic in relation to physical health: (a) moderate exercise, (b) strength training, and (c) sitting time. COVID-19: coronavirus disease 2019.

We also examined the associations of sex and pandemic with physical activity. Figure 1 shows the 3 measurements of physical activities in the male and female populations, before and after the pandemic.

Figure 1 (a) compares the minutes of moderate exercise per day, where outliers (minutes ≥200) are removed. The association with sex is significant for moderate exercise per day after the pandemic. Figure 1 (b) compares the frequency of strength training per week, and we see that males tend to have a higher frequency of strength training regardless of the pandemic. Figure 1 (c) compares the sitting time per day, showing that males sit slightly longer than females regardless of the pandemic. Both males and females had a slight decrease in terms of the quantity and intensity of physical activity. Females had a lower level of physical activity than males, and this gap slightly increased during the pandemic.

## DISCUSSION

Sex is a biological and physiologic trait that characterizes males and females; and gender, a continuum of socio-culturally constructed roles and behaviors associated with men, women, and gender-spectrum diversity, are among the most important determinants of health and disease outcomes. However, these fundamental factors are often ignored in biomedical research and are rarely incorporated into clinical care. Sex disparity in health care is a social phenomenon in which males and females do not receive care equally.^22^ The biological, behavioral, social, and systemic factors underlying the differences in how females and males may experience COVID-19 and its consequences cannot be oversimplified.^23^ The COVID-19 pandemic appears to have exacerbated the inequalities that already existed between males and females globally. There is a need to incorporate the sex- and gender-specific and differentiating factors into the research, prevention, and therapeutics implementation responses to the COVID-19 pandemic.^24^ Adopting sex- and gender-informed perspective in research has already shown evidence to improve patient care for health care outcomes that affect both females and males.^25^

Translating this perspective to health care research requires collecting large-scale sex- and gender-disaggregated data. This task may pose some methodological challenges for gender, given the lack of validated tools to assess it. This study utilized a nationally representative dataset to examine the association of the COVID-19 pandemic, the association of sex, and the joint association of sex and the COVID-19 pandemic with health and health care. We did not see a significant association of the interaction term for all the characteristics, suggesting that the pandemic has not affected male and female populations with statistically significant difference. However, the results showed that the pandemic amplified some existing sex differences in health information seeking, communication, sharing, physical activity, and mental health. To avoid perpetuating sex disparity and health inequities in responses to public health emergencies such as COVID-19, it is important that sex norms, roles, and relations that influence females’ and males’ differential health communication behaviors should be considered and addressed.^26^

Individuals seek information for informed decision-making, and they consult a variety of information sources nowadays. Early evidence showed mixed results regarding whether the widespread transition to telehealth during the pandemic was creating additional fractures in society by disproportionately influencing health equity.^5^ Our results show that the implementation of remote health care or using mobile health needs to be grounded in sex analysis and ensure meaningful participation of affected groups, including females. Because females were more likely to use mobile health and health communication technologies than males, and the difference increased after the pandemic. The health communication and social networking platforms could potentially provide effective strategies to provide new ways to facilitate health care delivery during the pandemic.^27^^,^^28^ We found that sex differences in using mobile health, wearable and portable devices,^29^ and social networking services and females were more likely to use these tools while the differences were not statistically amplified by the pandemic. The findings aligned with a prior study that reported large sex differences in COVID-19-related beliefs and behaviors, with women being more likely to perceive the pandemic as a serious health problem and to agree and comply with restraining measures.^30^ Further research is needed to understand the quantitative and dynamic patterns of the use of health communication tools to measure its benefits and harmful effects and inform evidence-based approaches to clinical interventions ,^31^ practices, policy, education, and regulation. Leveraging the social networking services and data-gathering functions of digital platforms in the right ways, we may achieve breakthroughs in the technologies’ ability to decrease sex-related disparities.^32^

The pandemic induces a slight decrease in the quantity and intensity of physical activity in both males and females, as well as an increase in alcohol drinking, which has a negative association with health. We found that females have a lower level of physical activity than males, and this gap has slightly increased during the pandemic. The association of sex is significant before and after the pandemic with alcohol assumption, and males drink more than females. The pandemic did not amplify the differences in alcohol intake between sexes. Sex-related differences in lifestyles and social roles require careful consideration as they are believed to greatly influence the onset, course, and outcomes of the COVID-19 pandemic.

In terms of mental health, the COVID-19 pandemic can be experienced in many different ways, including feelings of depression, fear, panic, and anxiety. Beyond being a pandemic infectious disease, COVID-19 also acts as a potent stressor, with millions of individuals experiencing fear and social isolation over a prolonged period.^16^ Exposure to persistent stress is associated with increased vulnerability to and severity of stress-related psychiatric disorders (such as posttraumatic stress disorder, panic disorder, and major depression), which occur more frequently in females than males.^33^ Stress-related disorders and the long-term consequences of COVID-19 on health outcomes highlight another important association with sex. A preliminary study showed that there was an increased prevalence and severity of depressive, anxious, and posttraumatic symptoms in females than in males.^34^ In this study, we also found that females were more likely to have increased vulnerability to and severity of stress-related psychiatric disorders than males while the pandemic did not significantly amplify that difference. This sex difference is also related to response systems,^35^ which increase endocrine, affective, and arousal responses to stress in females.^33^ Longitudinal studies are warranted to test whether the relationship between stress exposures and the prevalence and presentation of stress-related psychiatric disorders were mediated by sex- and gender-related factors. These observations may be helpful in developing and implementing prevention and treatment interventions that are able to address the acute and long-term effects of the pandemic on the health and well-being of both male and female populations.^36^

## LIMITATION AND FUTURE WORK

While the mailing and the reminder postcards were sent out on schedule and without any issues, the World Health Organization’s announcement on March 11 of the COVID-19 pandemic impacted the rest of the field period in HINTS 5 Cycle 4. Given the limitations of the dataset, we did not have information about when the participants filled out the survey, which may have impacted the results. Because the survey was cross-sectional, we could not examine causality among variables. The use of the legal sex as a binary variable was representative of the participant cohort but may not be inclusive. The lack of granularity on gender and gender minorities may also impact the generalizability. Future work may use sex when reporting biological factors, gender when reporting gender identity or sociocultural factors, and asking individuals about both their sex assigned at birth and their current gender identity; which may facilitate data collection, increase inclusiveness, and improve comparability across studies. Despite these limitations, this study provides nationally representative estimates and contributes to a better understanding of the impact of sex differences and the COVID-19 pandemic on common characteristics of health and health care.

## CONCLUSION

This study used multivariate models to understand the association of the COVID-19 pandemic, the association of sex, and the joint association of sex and the COVID-19 pandemic with health communication, physical activity, mental health, and behavioral health. The findings of this study demonstrate that the COVID-19 pandemic amplifies existing sex disparities in some health and health care domains. Intersectional analyses of sex are integral to addressing issues that arise and mitigating inequities. Responses to the pandemic should consider diverse perspectives, including sex and gender. It is essential to recognize and investigate sex-related effects on the COVID-19 pandemic to collect accurate data, and develop and implement prevention and treatment interventions to address the acute and long-term impacts of the pandemic on the health and well-being of different populations.

## AUTHOR CONTRIBUTIONS

JY conceived and designed the study, contributed to the analyses, and drafted the manuscript. ZR contributed to the analyses. JY and ZR contributed to the interpretation of the results, drafting, and revision of the manuscript. All the authors read and approved the final version of the manuscript.

## SUPPLEMENTARY MATERIAL

Supplementary material is available at *JAMIA Open* online.

## CONFLICT OF INTEREST STATEMENT

None declared.

## Supplementary Material

ooac076_Supplementary_Data

## Data Availability

All data referred to in the manuscript are available at: https://www.cancer.gov/ and the Dryad Digital Repository: doi:10.5061/dryad.h70rxwdn5.
